# Metastasis-Associated Cell Surface Oncoproteomics

**DOI:** 10.3389/fphar.2012.00192

**Published:** 2012-11-07

**Authors:** Piia-Riitta Karhemo, Maija Hyvönen, Pirjo Laakkonen

**Affiliations:** ^1^Research Programs Unit, Molecular Cancer Biology and Institute of Biomedicine, Biomedicum Helsinki, University of HelsinkiHelsinki, Finland

**Keywords:** metastasis, cell surface, proteomics, biotinylation, cancer

## Abstract

Oncoproteomics aims to the discovery of molecular markers, drug targets, and pathways by studying cancer specific protein expression, localization, modification, and interaction. Cell surface proteins play a central role in several pathological conditions, including cancer and its metastatic spread. However, cell surface proteins are underrepresented in proteomics analyses performed from the whole cell extracts due to their hydrophobicity and low abundance. Different methods have been developed to enrich and isolate the cell surface proteins to reduce sample complexity. Despite the method selected, the primary difficulty encountered is the solubilization of the hydrophobic transmembrane proteins from the lipid bilayer. This review focuses on proteomic analyses of metastasis-associated proteins identified using the cell surface biotinylation method. Interestingly, also certain intracellular proteins were identified from the cell surface samples. The function of these proteins at the cell surface might well differ from their function inside the cell.

## Role of Cell Surface Proteins in Cancer Progression and Metastasis

Cell surface forms a physical barrier between the cell and its environment simultaneously mediating multiple crucial signaling events. It is mainly composed of proteins that are embedded in the lipid bilayer of plasma membrane. These proteins can be classified as transmembrane proteins with one or multiple transmembrane domains, lipid anchored proteins, or peripheral membrane proteins that are attached to other cell surface proteins or peripheral regions of the lipid bilayer.

Cell surface molecules play an important role in mediating cell–cell interactions and signaling between the cells and between the cells and their environment. In general, cell surface proteins can be divided into ligand-gated ion channels, G-protein linked, enzyme-linked, and cell adhesion receptors (Kabbani, 2008). Especially G-protein and enzyme-linked receptors are important in sensing extracellular ligands, such as growth factors and hormones, which then participate in the complex intracellular signaling pathways. In addition, cell adhesion proteins are vital for the cell attachment to the extracellular matrix (ECM) components, such as collagens, laminins, and proteoglycans. Cell adhesion is also crucial for cell migration and communication between the neighboring cells.

Composition of the cell surface is dynamic and changes constantly due to the internalization, secretion, and shedding of proteins in response to internal and external stimuli. Cell surface undergoes modifications during different developmental stages and its structure varies between different cell types. Importantly, cell surface molecules play a central role in several pathological conditions, including cancer and its metastatic spread (Kawaguchi, 2005; Dowling et al., 2008).

A key event in cancer progression is the acquisition of an invasive phenotype that allows cancer to spread to distant organs in the body and form metastatic lesions. Metastatic dissemination, rather than the primary tumor, causes 90% of cancer deaths. Cancer metastasis can be divided into the following steps; detachment from the primary tumor, invasion of the lymphatics and/or blood vessels, transport to distant organs, extravasation, and proliferation at the secondary site (Fidler, 2003). This cascade is complicated by the fact that different tumor types metastasize to a different set of organs (Auerbach et al., 1987; Johnson et al., 1991; Nguyen et al., 2009). The importance of the cell surface in the metastasis has been recognized already during 1970s and 1980s (Davey et al., 1976; Pearlstein et al., 1980; Poste and Nicolson, 1980; Fogel et al., 1983). Cell surface proteins play an important role in the signaling and adhesive interactions between the tumor and stromal cells during the metastatic dissemination and during the organ specific homing of metastatic cells (Ruoslahti and Rajotte, 2000; Brown and Ruoslahti, 2004; Dowling et al., 2008; Brooks et al., 2010). Cell surface proteins are also optimal targets for cancer therapies due to their accessibility. Currently, while making only about 22% of all proteins encoded in the human genome, cell surface molecules represent two-thirds of the protein-based drug targets (Hopkins and Groom, 2002; Overington et al., 2006).

## Cell Surface Oncoproteomics

Oncoproteomics aims to the discovery of molecular markers, drug targets, and pathways by studying cancer specific protein expression, localization, modification, and interaction. As stated above, the cell surface plays a crucial role in cancer metastasis. Therefore, it is important to discover the differences between the surface of a normal or a non-metastatic cancer cell and a metastatic one. However, the poor solubility and low abundance of cell surface proteins lead to their under representation in proteomic studies performed from the whole cell extracts. With the aid of protein fractionation and more sensitive technologies, such as mass spectrometry based proteomics, researches have revealed novel proteins, and suggested new functions for known proteins. Different methods have been developed to isolate and enrich the cell surface and/or plasma membrane proteins for proteomic analyses (recently reviewed in Elschenbroich et al., 2010). However, it is important to notice that the cell surface and plasma membrane fractions are not completely identical. Ligands bound to their receptors, for example, can be considered as cell surface proteins but not as true plasma membrane proteins due to their lack of direct contact with it. In addition, during the preparation of a plasma membrane fraction, intracellular proteins attached to the plasma membrane are also isolated. These, however, cannot be considered cell surface proteins.

**Figure 1:**
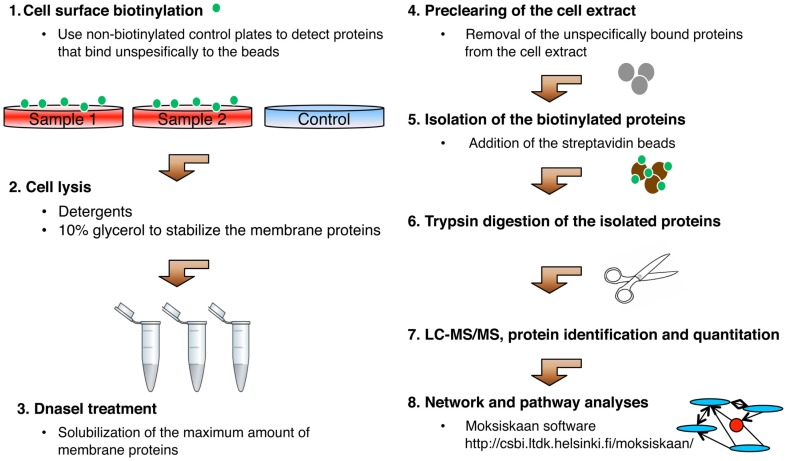
**Schematic representation of the optimized isolation of biotinylated cell surface proteins**.

The most commonly used method for the isolation of plasma membrane fraction is homogenization combined with membrane density separation (Elschenbroich et al., 2010; Leth-Larsen et al., 2010). Plasma membrane proteins can also be isolated by the electrostatic attachment of cationic colloidal silica to the membranes (Rahbar and Fenselau, 2004) combined with Triton X-114 phase partitioning (Mathias et al., 2011). As most secreted and cell surface proteins are glycosylated, they can be isolated with the aid of the cell surface capture technique (CSC) after labeling of the glycan moieties (Wollscheid et al., 2009), with a lectin affinity approach (Wang et al., 2008), and with metabolic labeling of the glycans followed by affinity isolation (Yang et al., 2011). Due to their accessibility, cell surface proteins can easily be labeled with a membrane-impermeable biotin and isolated with streptavidin-linked beads from the cell extracts (Elia, 2008). We have recently optimized the isolation of biotinylated cell surface proteins (Karhemo et al., 2012). The flow chart of the optimized method is shown in Figure 1. Using the labeling methods, all proteins accessible for the labeling reagent e.g., ligands bound to their receptors are isolated and analyzed with downstream applications. When adherent cell cultures are used as a starting material secreted and ECM proteins can also be isolated. Despite the isolation method of the cell surface proteins, the primary difficulty encountered is the solubilization of the hydrophobic transmembrane proteins from the lipid bilayer. This is performed by the aid of different detergents, which should be chosen carefully to ensure that they do not interfere with the downstream applications. The use of detergents in membrane protein analyses has been recently reviewed (Seddon et al., 2004). Importantly, in the affinity enrichment of cell surface proteins with biotinylation or other labeling methods, only the proteins soluble in the detergent used will be isolated. To obtain the most comprehensive picture of cell surface proteins multiple isolations with different detergents are needed.

Following isolation cell surface proteins are analyzed, identified, and quantified with proteomics methods. Two dimensional (2D) gel electrophoresis is a conventional proteomics tool where proteins are first separated according to their isoelectric point (isoelectric focusing, IEF). The second dimension is an SDS-PAGE gel, which is stained to visualize the protein spots. The 2D-gel electrophoresis has very high resolution and it can separate post-translational variants of a single protein. With this high resolution one can assume that one protein spot represents a single protein and changes in the spot volume can be used to quantitate the expression differences between the analyzed samples. The proteins within interesting spots can be in-gel digested and identified using mass spectrometry. Hydrophobic membrane proteins, however, often precipitate at their isoelectric point in the first dimension. Therefore they do not enter the second dimension gel and are lost from the analyses. The use of 2D-electrophoresis to analyze membrane proteins has been recently reviewed (Rabilloud et al., 2008).

Shotgun proteomics is a method used to identify proteins from complex mixtures with high performance liquid chromatography combined with mass spectrometry. This is the most common method, with or without separation of the samples in an SDS-PAGE gel, used for identification of proteins from cell surface fractions. To create a quantitative method, various label-based and label-free approaches have been developed for the quantitation of differentially expressed proteins from gel free analyses (Coombs, 2011).

## Metastasis-Associated Cell Surface Proteins Identified Using Proteomics

To identify proteins essential for cancer metastasis, researchers have often compared protein expression between closely related or isogenic metastatic and non-metastatic cells. The use of isogenic cell pairs diminishes the genetic variation. Thus, the differential expression of a protein is more likely to be associated with the metastatic behavior than when using non-isogenic cell lines. Table 1 shows a selection of metastasis-associated proteins identified from different models with comparative cell surface oncoproteomics using either the cell surface biotinylation (Conn et al., 2008; Roesli et al., 2009; Luque-Garcia et al., 2010; Karhemo et al., 2012) or the plasma membrane isolation method (Leth-Larsen et al., 2009). We selected proteins to the Table 1 only if the differential expression and the cell surface localization of non-conventional cell surface proteins were validated in these studies.

**Table 1:** **Selection of metastasis-associated proteins identified with comparative cell surface oncoproteomics**.

Protein	Correlation of expression with metastasis	Cancer type	Cell lines/tissue used	Metastatic site	Isolation method	Detergent used in isolation of biotinylated proteins	Quantification method	Differential expression validated	Reference
*Actn*	Up	Murine terato-carcinoma	F9B9, F9DR	Liver	a	*	Two dimensional peptide mapping (Spectational software)	IF (cells)	Roesli et al. (2009)
*Hmgb1*	Up	Murine terato-carcinoma	F9B9, F9DR	Liver	a	*	Two dimensional peptide mapping (Spectational software)	IF (cells) also surface expressed *in vivo*	Roesli et al. (2009)
*Ak2*	Up	Murine terato-carcinoma	F9B9, F9DR	Liver	a	*	Two dimensional peptide mapping (Spectational software)	IF (cells)	Roesli et al. (2009)
*Syk*	Down	Murine terato-carcinoma	F9B9, F9DR	Liver	a	*	Two dimensional peptide mapping (Spectational software)	IF (cells) also surface expressed *in vivo*	Roesli et al. (2009)
*Dpp4*	Down	Murine terato-carcinoma	F9B9, F9DR	Liver	a	*	Two dimensional peptide mapping (Spectational software)	IF (cells)	Roesli et al. (2009)
*Crry*	Down	Murine terato-carcinoma	F9B9, F9DR	Liver	a	*	Two dimensional peptide mapping (Spectational software)	IF (cells)	Roesli et al. (2009)
*CDH17*	Up	Colorectal cancer	KM12SM, KM12C	Liver	a	**	SILAC	WB (total extracts), FACS, IF (cells), IHC (patients)	Luque-Garcia et al. (2010)
*JUP*	Up	Colorectal cancer	KM12SM, KM12C	Liver	a	**	SILAC	WB (total extracts), IHC (patients)	Luque-Garcia et al. (2010)
*CEACAM5*	Up	Colorectal cancer	KM12SM, KM12C	Liver	a	**	SILAC	WB (total extracts)	Luque-Garcia et al. (2010)
*EGFR*	Up	Colorectal cancer	KM12SM, KM12C	Liver	a	**	SILAC	WB (total extracts), IHC (patients)	Luque-Garcia et al. (2010)
*TIMP-2*	Down	Fibrosarcoma	HT-1080 variants	Lungs	a	***	Spectral counting	WB (surface, total extracts, xenografts)	Conn et al. (2008)
*NCAM*	Up	Fibrosarcoma	HT-1080 variants	Lungs	a	***	Spectral counting	WB (surface)	Conn et al. (2008)
*JAM-C*	Up	Fibrosarcoma	HT-1080 variants	Lungs	a	***	Spectral counting	WB (surface, xenografts)	Conn et al. (2008)
*TF*	Up	Fibrosarcoma	HT-1080 variants	Lungs	a	***	Spectral counting	WB (surface, xenografts)	Conn et al. (2008)
*PROCR*	Up	MDA-MB-435	NM-2C5 M-4A4	Lungs	a	****	Ion intensities recorded in MS data (Progenesis LC-MS software)	WB (surface, total extracts) IF (cell surface), IHC (xenografts)	Karhemo et al. (2012)
*CD109*	Up	MDA-MB-435	NM-2C5 M-4A4	Lungs	a	****	Ion intensities recorded in MS data (Progenesis LC-MS software)	WB (surface, total extracts) IF (cell surface), IHC (xenografts)	Karhemo et al. (2012)
*ITGA6*	Up	MDA-MB-435	NM-2C5 M-4A4	Lungs	a	****	Ion intensities recorded in MS data (Progenesis LC-MS software)	IF (cell surface, xenografts)	Karhemo et al. (2012)
*PTGFRN*	Up	MDA-MB-435	NM-2C5 M-4A4	Lungs	a	****	Ion intensities recorded in MS data (Progenesis LC-MS software)	IHC (xenografts)	Karhemo et al. (2012)
*ART3*	Up	MDA-MB-435	NM-2C5 M-4A4	Lungs	a	****	Ion intensities recorded in MS data (Progenesis LC-MS software)	mRNA	Karhemo et al. (2012)
*NdrgI*	Up	MDA-MB-435	NM-2C5, M-4A4	Lungs	b		SILAC	IHC (cells, patients)	Leth-Larsen et al. (2009)
*HLA-DR*α	Up	MDA-MB-435	NM-2C5, M-4A4	Lungs	b		SILAC	WB (PM) IHC (cells)	Leth-Larsen et al. (2009)
*HLA-DR*β	Up	MDA-MB-435	NM-2C5, M-4A4	Lungs	b		SILAC	WB (PM) FACS, IHC (cells, patients)	Leth-Larsen et al. (2009)
*CD74*	Up	MDA-MB-435	NM-2C5, M-4A4	Lungs	b		SILAC	FACS, IHC (cells)	Leth-Larsen et al. (2009)
*ecto-5’-NT*	Up	MDA-MB-435	NM-2C5 M-4A4	Lungs	b		SILAC	WB (PM) FACS, IHC (patients)	Leth-Larsen et al. (2009)
*CD44v6*	Down	MDA-MB-435	NM-2C5 M-4A4	Lungs	b		SILAC	FACS, IHC (cells)	Leth-Larsen et al. (2009)

Isolation methods: a. Cell surface biotinylation. b. Centrifugation and Percoll-sucrose density separation. Detergents: *2% (w/v) NP40 substitute (Fluka), 0.2% (w/v) SDS.

**Cell surface protein isolation kit (Pierce). ***Octyl-β-d-glucopyranoside buffer. ****10% glycerol, NP40, and Triton-X100. Abbreviations: WB, Western Blot; PM, plasma membrane; IF, Immunofluorescence; FACS, Fluorescence-Activated Cell Sorting; IHC, Immunohistochemistry; mRNA, messenger RNA.

In addition to the identifications of known players in metastasis novel candidate metastasis-associated proteins were identified in these studies. Some of these candidates are conventionally considered to reside inside the cell and might represent contamination of the cell surface fraction with the intracellular proteins. However, the cell surface expression of some of the intracellular proteins was confirmed by other methods in these studies. The cell surface localization of conventional intracellular proteins has also been reported in other studies (rewieved in Butler and Overall, 2009). For an unknown reason tumor cells appear to display extracellularly proteins that normally reside inside the cell. The function of an intracellular protein at the cell surface might well differ from its function inside the cell. The cell surface function of these non-classical cell surface proteins might also be affected by post-translational modifications that change their physical properties, solubility, localization, and interactions with other proteins.

Below we will review some known and novel metastasis-associated proteins identified using the comparative cell surface proteomics. Interestingly, only minor overlap exists between proteins identified in different experimental models or even when the same model has been analyzed using different methods for the sample preparation, antigen detection, and protein quantification. Only three common proteins were identified (HLA-DRB1, HLA-DRA1, and ITGA6) in two studies analyzing the cell surface proteins of a non-metastatic and metastatic pair of human breast cancer/melanoma cell line MDA-MB-435 either using the plasma membrane isolation (Leth-Larsen et al., 2009) or the cell surface biotinylation method (Karhemo et al., 2012). Comparison of different studies is complicated by the fact that one protein might have multiple names or different accession numbers in different databases. In the future, it would be interesting to analyze the molecular pathways and interactomes of all identified and validated metastasis-associated cell surface proteins in a bioinformatics platform. In this review, the comparison was performed manually for a short overview. Interestingly, upregulation of different forms of protein tyrosine phosphatases (PTPs) have been identified on the surface of the metastatic cells in three of the studies analyzed (Conn et al., 2008; Roesli et al., 2009; Karhemo et al., 2012). PTPs can either promote or suppress tumor progression and metastasis via either enhancing or suppressing cell surface receptor tyrosine kinase signaling (reviewed in Sastry and Elferink, 2011).

The junction plakoglobin (JUP, catenin gamma), reported to be overexpressed in the metastatic colorectal cancer cells (Luque-Garcia et al., 2010), was found to be downregulated in the highly invasive and metastatic fibrosarcoma cells (Conn et al., 2008). The differential expression was not, however, validated in the fibrosarcoma model and the Western blot validation performed in the colorectal cancer showed only 1.4-fold higher expression in the metastatic cell line. According to immunohistochemical analysis of colorectal cancer specific tissue microarrays the expression of JUP was higher in the epithelial cells of tumor tissue than in their normal counter parts. In addition, most of the late stage tumors showed a clear overexpression of the protein (Luque-Garcia et al., 2010). The contradictory results in these studies could be explained by the different origin of the tumor cell lines and by the different localization of the metastatic site (liver vs. lungs). According to a recent review on the function of JUP in cancer and metastasis it appears generally act as a tumor/metastasis suppressor protein (Aktary and Pasdar, 2012).

The ecto-5′-nucleotidase (5′-nucleotidase, ecto-5′-NT, CD73), overexpressed in the metastatic variant of the MDA-MB-435 cells (Leth-Larsen et al., 2009) has been shown to affect tumor growth by limiting the antitumor T-cell immunity via adenosine receptor signaling. Adenosine is an important metabolite released by cancer cells to establish physiological conditions conducive for tumorigenesis (Spychala, 2000). Moreover, it has been reported that a small interfering RNA (siRNA) against the ecto-5′-NT effectively inhibits invasion and migration of the highly aggressive human MDA-MB-231 breast cancer cells and prevents their adhesion to the ECM (Zhi et al., 2007). The role of ecto-5′-NT as a promising target for tumor therapy has been recently discussed (Zhang, 2010).

Roesli et al. (2009) reported overexpression of the adenylate kinase 2 (Ak2, Adk2) on the surface of the metastatic F9DR murine terato-carcinoma cells compared to the non-metastatic F9B9 cell line. Based on the microarray data Ak2 is also upregulated in the metastatic pancreatic endocrine neoplasms (Hansel et al., 2004). Ak2 is thought to be a mitochondrial protein that catalyzes a reversible transfer of the terminal phosphate group between ATP and AMP (Dzeja et al., 1998) and plays a key role in hematopoiesis. In addition, Ak2 is involved in energy metabolism and nucleotide synthesis (Lagresle-Peyrou et al., 2009). Roesli et al. (2009), however, confirmed that Ak2 localized on the cell surface of the metastatic cells by analyzing the optical sections of the confocal microscopic images. Interestingly, Ak2 has been shown to play a role in bovine sperm flagella movement (Schoff et al., 1989). Further studies are needed to reveal the function of Ak2 at the cell surface of the metastatic cells.

The junctional adhesion molecule C (JAM-C) was reported to be overexpressed on the surface of the invasive HT-1080 fibrosarcoma cells as compared to its non-invasive counterpart (Conn et al., 2008). Overexpression of JAM-C in the invasive HT-1080 cells also significantly decreased the life span of tumor-bearing mice (Fuse et al., 2007). JAM-C, together with JAM-A and B, belongs to the immunoglobulin subfamily and is expressed by leukocytes and platelets as well as by epithelial and endothelial cells. It localizes to cell-cell contacts and is specifically enriched at tight junctions making it an interesting candidate for functional studies in metastasis. A more detailed description of junctional adhesion molecules can be found in Ebnet et al. (2004) and Bradfield et al. (2007).

We recently identified CD109, PTGFRN, and ART3 as novel metastasis-associated candidate proteins (Karhemo et al., 2012). CD109 is a GPI-linked cell surface protein expressed on the surface of activated platelets (Smith et al., 1995). It negatively regulates TGFB1 signaling in keratinocytes (Finnson et al., 2006; Hagiwara et al., 2010). Shedding of CD109 by mesotrypsin has been shown to be important for the malignant growth of breast cancer cells in a three dimensional organotypic cell culture (Hockla et al., 2010). In addition, expression of its transcript has been linked to melanoma in a transgenic melanoma mouse model (Ohshima et al., 2010). However, the function of CD109 in invasion and metastasis is unknown. Expression of PTGFRN positively correlates with the metastatic status of human lung tumors (Guilmain et al., 2011). PTGFRN associates with and inhibits the binding of prostaglandin F2α to its receptor (Orlicky, 1996). It also participates in the tetraspanin web (Charrin et al., 2001, 2003; Stipp et al., 2001; Guilmain et al., 2011). The tetraspanin web proteins either promote or suppress tumor invasion and metastasis depending on the composition of the tetraspanin-enriched microdomain on the cell surface (Richardson et al., 2011). The function of PTGFRN in the tetraspanin web is unknown. Based on its overexpression on the metastatic cell line, it might participate in functions promoting metastasis. Not much is known about the function of ART3 (Ecto-ADP-ribosyltransferase 3), which hampers its functional analysis in metastasis. Due to the lack of proper antibodies against ART3, we were only able to validate its overexpression in the metastatic cell line at the mRNA level (Karhemo et al., 2012). Ecto-ADP-ribosyltransferases are a group of cell surface enzymes that reversible transfer ADP-ribose groups onto target proteins thus modifying their function. The ADP-ribosylation of cell surface proteins provides sophisticated regulatory mechanisms for cell communication making ART3 as an interesting target in functional studies of metastasis.

## *In vivo* Validation of the Results

As stated in the previous sections, composition of the cell surface changes constantly in response to environmental stimuli. When tumor cells are cultured *in vitro* they lack the 3-dimensional microenvironment and contacts with stromal cells that the metastatic tumor cell encounters while growing both at the primary and secondary sites. For these reasons, it is important to further validate the obtained *in vitro* results using *in vivo* models. As seen in Table 1 the differential expression of some of the identified proteins was validated *in vivo* either in xenograft tumors derived from the same cells that were used in the proteomics study or in patient material. In addition to the *in vivo* validation, it is as important to perform functional assays to reveal mechanisms underlying the differential expression and the role of the identified proteins in the metastatic process. From the studies reviewed here, only the function of tissue factor was validated in invasion and metastasis (Conn et al., 2008). In the future it might be important to focus on the identified metastasis-associated cell surface proteins to reveal their interacting partners and pathways they are involved in. Moreover, significance of the cell surface localization of the intracellular proteins needs to be further studied.

In order to discover metastasis-associated endothelial markers *in vivo* biotinylation of the vasculature of liver metastasis was performed (Borgia et al., 2010). Methods that would allow analysis of cell surface proteomes of primary tumors and their matched metastases from paraffin embedded tissue samples would also greatly facilitate the identification of *in vivo* contributors and possible drug targets. In addition, it would be beneficial to analyze the expression changes of stromal cell surface proteins at the secondary sites in response to metastatic tumor cells (Garin-Chesa et al., 1990; Huang et al., 2005).

## Concluding Remarks

Reduction of sample complexity by cell fractionation and subcellular proteome analysis allows an in-depth analysis of intermediate and low abundant proteins. Cell surface biotinylation is an excellent method to identify known and novel metastasis-associated cell surface proteins from various models. In addition, it can be used to discover novel localizations for already known proteins. When using the cell surface biotinylation method, selection of detergents is of utmost importance and influences the type of proteins that will be identified with the downstream applications. The interesting novel metastasis-associated protein candidates identified need further functional validation to resolve their role in metastatic spread of tumors and possible use as novel drug targets.

## Conflict of Interest Statement

The authors declare that the research was conducted in the absence of any commercial or financial relationships that could be construed as a potential conflict of interest.
